# Wide-Range Probing of Dzyaloshinskii–Moriya Interaction

**DOI:** 10.1038/srep45498

**Published:** 2017-03-31

**Authors:** Duck-Ho Kim, Sang-Cheol Yoo, Dae-Yun Kim, Byoung-Chul Min, Sug-Bong Choe

**Affiliations:** 1 Department of Physics and Institute of Applied Physics, Seoul National University, Seoul, 08826, Republic of Korea; 2 Center for Spintronics, Korea Institute of Science and Technology, Seoul, 02792, Republic of Korea

## Abstract

The Dzyaloshinskii–Moriya interaction (DMI) in magnetic objects is of enormous interest, because it generates built-in chirality of magnetic domain walls (DWs) and topologically protected skyrmions, leading to efficient motion driven by spin–orbit torques. Because of its importance for both potential applications and fundamental research, many experimental efforts have been devoted to DMI investigation. However, current experimental probing techniques cover only limited ranges of the DMI strength and have specific sample requirements. Thus, there are no versatile methods to quantify DMI over a wide range of values. Here, we present such an experimental scheme, which is based on the angular dependence of asymmetric DW motion. This method can be used to determine values of DMI much larger than the maximum strength of the external magnetic field strength, which demonstrates that various DMI strengths can be quantified with a single measurement setup. This scheme may thus prove essential to DMI-related emerging fields in nanotechnology.

The Dzyaloshinskii–Moriya interaction (DMI) is an antisymmetric exchange interaction that occurs at interfaces between ferromagnetic and heavy metal layers with large spin–orbit coupling^1^,^2^,^3^. In magnetic systems, DMI generates chiral spin textures such as Néel domain walls (DWs)^4^,^5^,^6^,^7^ and magnetic skyrmions^8^,^9^,^10^. Because these chiral spin textures promise several potential applications^4^,^7^,^8^, it is crucial to quantify the strength of the DMI both to better understand its physical origin and for the technical optimization of ferromagnetic materials.

Several experimental schemes have been proposed to measure the DMI strength^5^,^6^,^11^,^12^,^13^,^14^,^15^. Using an optical microscope, it has been developed a method to estimate it based on the field-driven asymmetric DW speed with respect to an in-plane magnetic field^11^,^16^,^17^ and the current-driven asymmetric DW speed^5^,^6^. Moon *et al*.^12^ suggested another approach based on the frequency nonreciprocity, which provides a way to measure the DMI constant. Other measurement schemes relying on the nonreciprocal propagation of spin waves were demonstrated using Brillouin light scattering (BLS)^18^,^19^,^20^ and inductive ferromagnetic resonance (FMR)^21^. All these techniques are applicable to different ranges of DMI strength and have different sample requirements.

The optical microscopy technique^11^,^16^,^17^ based on asymmetric DW speed provides an easy and direct way to measure DMI-induced effective magnetic fields. However, its measurement range is limited by the maximum strength of the external in-plane magnetic field, which in turn is fundamentally constrained by the narrow space available inside the optical setup. Moreover, application of large external magnetic fields to the optical setup requires sophisticated care to prevent artifacts caused by stray fields from electromagnets as well as mechanical, optical, and thermal artifacts from such strong magnetic fields. In this study, we propose a way to overcome this field strength limit by utilizing DWs, inclined at an angle with respect to the direction of the in-plane magnetic field.

## Results

### DW energy model for a DW at an angle *θ*

**Figure 1:**
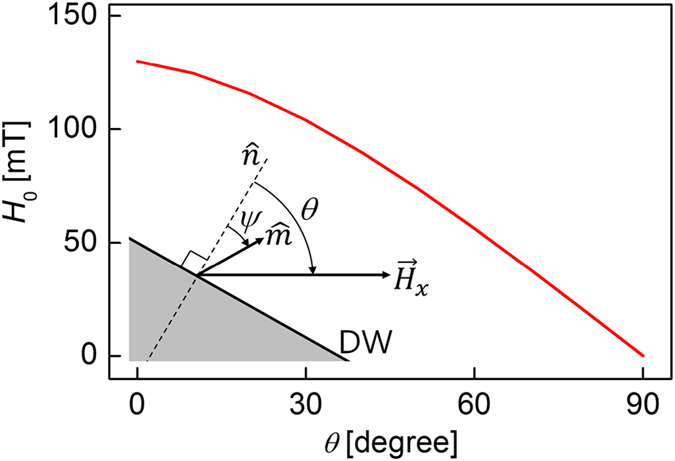
Plot of *H*_0_ as a function of *θ*. The Red solid line is calculated by using Eq. (4). The inset is an schematic illustration of the measurement geometry.

The inset in Fig. 1 shows the case where a DW is placed at an angle *θ* with respect to an in-plane magnetic field, *H*_*x*_. The DW energy density σ_DW_ can be expressed as a function of *H*_*x*_ and the angle *ψ* between the magnetization direction and the normal to the DW^3^,^11^:


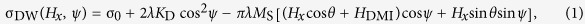


where σ_0_ is the Bloch-type DW energy density, *λ* is the DW width, *K*_D_ is the DW-anisotropy energy density, *M*_S_ is the saturation magnetization, and *H*_DMI_ is the DMI-induced effective magnetic field in the direction normal to the DW. The second term in the right-hand side of the equation corresponds to the DW-anisotropy energy and the third term to the Zeeman energy, including the DMI as an effective magnetic field. Note that Eq. (1) is identical to the Stoner–Wohlfarth equation^22^ for torque magnetometry with an additional unidirectional bias from *H*_DMI_.

For a given *H*_*x*_, the equilibrium angle *ψ*_eq_ can be obtained by the minimization condition 
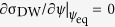
. Moreover, a numerical analysis of Eq. (1) shows that σ_DW_ has a maximum at *H*_*x*_ = *H*_0_, where *H*_0_ can be obtained from the maximization condition 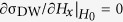. By solving these minimization and maximization conditions simultaneously, one can readily obtain two coupled equations:


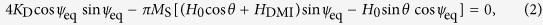



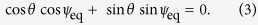


Equation (3) is identical to the relation 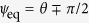, which implies that the DW magnetization stays perpendicular to the direction of *H*_0_. Inserting this value of *ψ*_eq_ into Eq. (2), it can be rewritten as


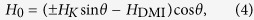


where *H*_K_ (≡4*K*_D_/*πM*_S_) is the DW-anisotropy field, which usually is small. Hence, in practice, the sign of the first term on the right-hand side of Eq. (4) coincides with that of *H*_DMI_, i.e., a plus sign for a positive *H*_DMI_ and a minus sign for a negative *H*_DMI_. Note that the well-known relation *H*_0_ = −*H*_DMI_^11^,^16^,^17^ can be restored in the limit *θ* → 0. Figure 1 plots the value of *H*_0_(*θ*) obtained from Eq. (4). This plot shows that *H*_0_ has the clear angle dependence.

Equation (4) contains the key idea of this study: one can significantly reduce the value of *H*_0_ by increasing *θ*. With this scheme, the magnitude of *H*_0_ can be adjusted down to a small experimental range *H*_range_ of the external magnetic field, which allows one to measure a large *H*_DMI_ without upgrading the electromagnet. For example, by tilting the DWs up to about 80°, one can measure *H*_DMI_ up to 1 T using an electromagnet with *H*_range_ ~ 200 mT, which is easily achieved in conventional optical setups^23^. It is also worth noting that by using this approach we can prevent a number of artifacts caused by large magnetic fields, such as mechanical instability produced by the induced magnetic moment in the optical setup, magneto-optical effects in the objective lens, and large Joule heating caused by the huge currents passing through the electromagnet.

### Verification of *θ*-dependence in Pt/Co/AlO_x_ films

**Figure 2:**
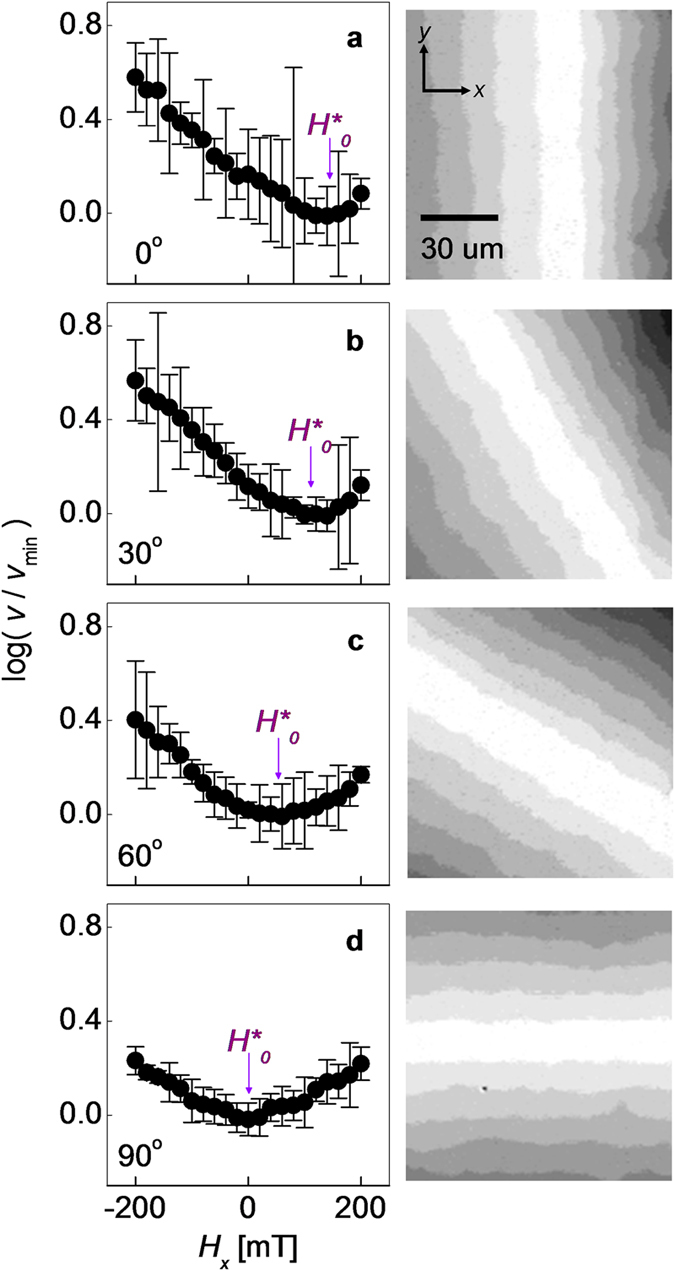
Plot of *v/v*_min_ as a function of *H*_*x*_ in a Pt/Co/AlO_x_ film, for fixed *H*_*z*_ = 5.5 mT and under various values of *θ*. (**a**) 0° (**b**) 30° (**c**) 60°, and (**d**) 90°. The displacement of the DWs at each angle is driven by the external magnetic fields.

To verify the feasibility of the present scheme, it was applied to ferromagnetic Pt/Co/AlO_x_ films, for which *H*_DMI_ is slightly smaller than *H*_range_. The procedure to measure *H*_0_ closely follows ref. 11, except for the initially tilted DWs. The tilted DWs were generated using a thermomagnetic writing technique^7^,^24^,^25^ (see Methods). The images in the right panel of Fig. 2 show the displacements of the DWs for various values of *θ* with respect to the direction of in-plane magnetic field *H*_*x*_ (=120 mT) under the application of a fixed out-of-plane magnetic field *H*_*z*_ (=5.5 mT) bias. Each image was obtained by adding several images, sequentially acquired during the DW displacement with a constant time step (=500 ms). Thus, each image simultaneously shows several DWs moving from brighter to darker interfaces as time goes by. One can then measure the DW speed *v* for each image. The plots in the left panel of Fig. 2 show the normalized DW speed *v/v*_min_ in the direction normal to the DW as a function of *H*_*x*_ (in creep regime), where *v*_min_ is the apparent minimum of *v*. It can be seen that *v(H*_*x*_) is symmetric under inversion with respect to 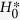 as shown in each plot. Here, 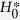 indicates the inversion symmetry axis where *v* has a minimum. According to ref. 11, *v* follows the creep relation 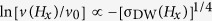, where *v*_0_ is the characteristic speed. In the case of clear inversion symmetry with a constant *v*_0_, the experimental 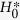 exactly matches *H*_0_, and thus, we will denote 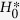 by *H*_0_ hereafter.

**Figure 3:**
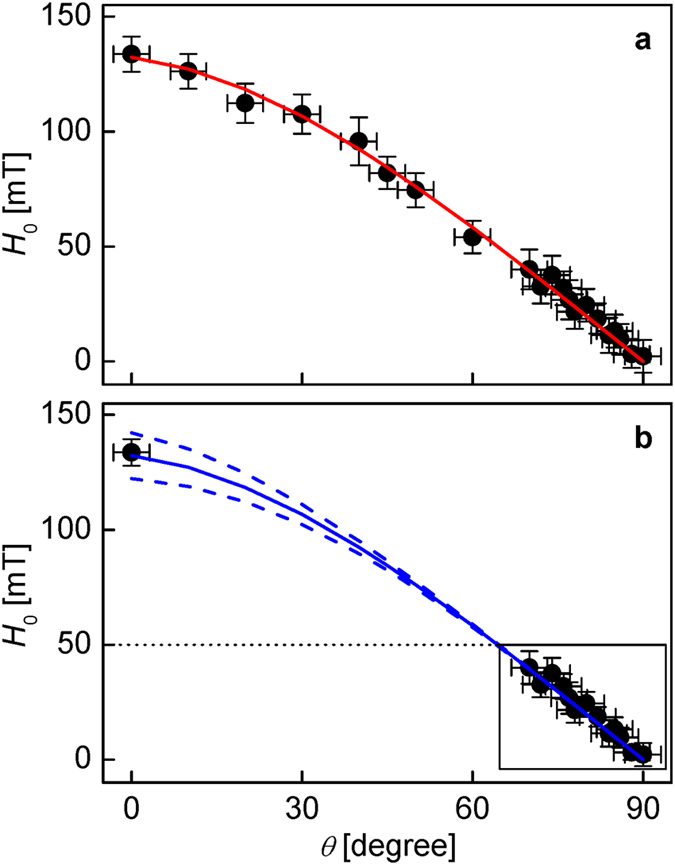
Plot of the measured *H*_0_ as a function of *θ* in a Pt/Co/AlO_x_ film. (**a**) Data collected for large *θ* range (from 0° to 90°). The red solid line shows the best fit to Eq. (4). (**b**) Data collected within a small *θ* range (from 70° to 90°). The blue solid line represents the best fit to Eq. (4) with the value of *H*_K_ fixed. The blue dotted lines in (**b**) are the best fits for the cases with *δH*_K_ =±10 mT.

Figure 3a plots the measured *H*_0_ as a function of *θ*. The red solid line shows the best fit to Eq. (4). The good agreement between the data and the fitting curve supports again the validity of the equation. The best-fit *H*_DMI_ (=−132 ± 3 mT) matches well the experimental value (=−134 ± 6 mT) measured at *θ* = 0. Moreover, the best-fit value of *H*_K_ (=−18 ± 5 mT) falls within the range of previous experimental reports^11^,^26^,^27^. The value of *H*_K_ can be alternatively measured through independent measurements^11^,^26^,^27^ or estimated using the relation 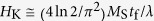
28,29, where *t*_f_ is the thickness of the magnetic layer.

### Application of present scheme to Pt/Co/AlO_x_ and Pt/Co/MgO films

To reproduce a situation in which *H*_range_ is limited (<50 mT), the fit was also performed only for the data (box in the plot) with large *θ* (≥70°) as shown in Fig. 3b. The blue solid line indicates the best fit to Eq. (4), using the fixed value of *H*_K_ obtained from Fig. 3a. This approach gives the best-fit value *H*_DMI_ (=−138 ± 12 mT), which again matches the previous values within the experimental accuracy. It is therefore demonstrated that the present approach enables one to measure large *H*_DMI_ in an experiment with limited *H*_range_. Note that the determined *H*_DMI_ is more than twice larger than *H*_range_.

Because the fit in Fig. 3b was performed with a fixed *H*_K_, now we examine the effect of the inaccuracy *δH*_K_ on *H*_K_. The blue dotted lines in Fig. 3b are the best fits when *δH*_K_ =±10 mT. The error *δH*_DMI_ is found to be slightly smaller than *δH*_K_, as expected from the relation *δH*_DMI_ = *δH*_K_sin*θ* deduced from Eq. (4). Because *H*_K_ is commonly within the range of a few tens of mT^11^,^26^,^27^,^28^,^30^, *δH*_K_ typically will not exceed about ±10 mT, and thus one can confirm that the error induced by *δH*_K_ error is not significantly large as compared to other experimental errors. Moreover, this error becomes negligible in practical cases because the present approach is designed for the determination of large *H*_DMI_ (≫*δH*_K_), significantly beyond the experimental *H*_range_.

**Figure 4:**
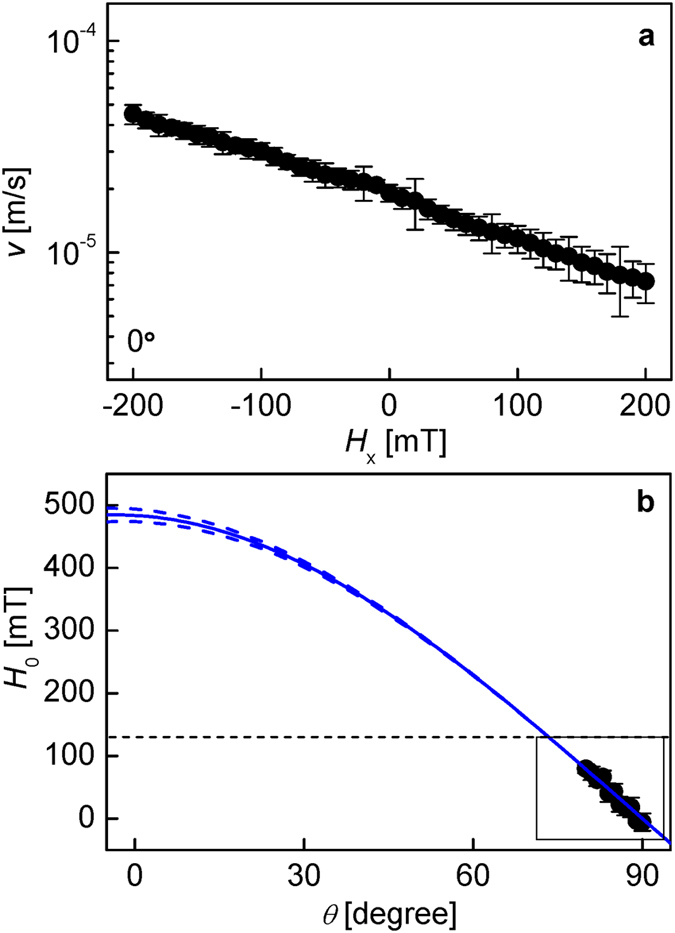
DMI determination in Pt/Co/MgO film. (**a**) Plot of the measured *v* as a function of the in-plane field *H*_*x*_, for fixed *H*_*z*_ = 20 mT, at *θ* = 0. (**b**) Plot of the measured *H*_0_ as a function of *θ*, for values of *θ* between 80° and 90°. The blue solid line shows the best fit to Eq. (4) and the blue dotted lines in (**b**) are the best fits for the cases with *δH*_K_ =±10 mT.

Finally, the present scheme was applied to Pt/Co/MgO films, which exhibit DMI larger than *H*_range_. Figure 4a shows *v* as a function of *H*_*x*_ for *θ* = 0. From this plot, it is apparent that the inversion symmetry axis *H*_0_ lies far beyond the experimental *H*_range_ (i.e., *H*_0_ ≫ 200 mT), and thus conventional optical schemes cannot be used to quantify *H*_DMI_. However, by applying the present method, Fig. 4b shows the measured *H*_0_ with respect to *θ* for large *θ* (≥80°). The black box in the figure indicates the measurable window for *H*_range_ in the present setup. The best fit (blue solid line) of *H*_K_ (=−30 ± 5 mT) indicates that *H*_DMI_ = −483 ± 10 mT, which is more than twice larger than *H*_range_. The sign and magnitude are in good agreement with previously reported results^21^. The blue dotted lines in Fig. 4b are the best fits for the cases with *δH*_K_ =±10 mT, and thus it is clearly demonstrated that the error becomes negligible in this case.

## Discussion

Additional asymmetry from chiral damping^31^ or asymmetric DW width variation^28^ may cause a shift *δH*_0_ in *H*_0_. However, because the asymmetric slope in *v* caused by these phenomena appears only during chirality variation occurring within the range of ±*H*_K_, |*δH*_0_| is essentially smaller than |*H*_K_|. Therefore, *δH*_0_-induced errors are negligible again in practice for large *H*_DMI_ determination.

In conclusion, we proposed a scheme to measure *H*_DMI_ over a wide range of values, overcoming the limitations caused by the small strength (*H*_range_) of the external magnetic fields typically used in experiments. By measuring the angular dependence of asymmetric DW motion, we found that *H*_0_ is strongly correlated with *θ*, which means that large DMI can be quantified in a robust manner by setting large values of *θ*. The feasibility of the present approach is experimentally demonstrated for various DMI strengths using ferromagnetic Pt/Co/AlO_x_ and Pt/Co/MgO films. The errors caused by additional asymmetry and inaccuracy of *H*_K_ were found to be negligible in practice for large *H*_DMI_ determination. The present scheme enhances the experimental range of optical measurement techniques without the need upgrade electromagnets. Our findings represent a novel and straightforward way to explore materials and systems with large DMI, and thus surmounts the key obstacle to design new devices in which the DMI is tailored to achieve for topological stability and efficient manipulation, as required for next-generation nanotechnology.

## Methods

### Sample preparation

For this study, we prepared 5.0-nm Ta/3.0-nm Pt/0.6-nm Co/1.6-nm AlO_x_ and 5.0-nm Ta/3.0-nm Pt/0.6-nm Co/2.0-nm MgO films, which were deposited on a Si wafer with a 100 nm SiO_2_ layer by dc magnetron sputtering^23^. To enhance the sharpness of the layer interfaces, we set a small deposition rate (=0.25 Å/s) through adjustment of the Ar sputtering pressure (~2 mTorr) and power (~10 W). All the films exhibited strong perpendicular magnetic anisotropy and circular domain expansion with weak pinning strength.

### Thermomagnetic writing of tilted domain walls

To create tilted DWs, we adopted a thermomagnetic writing technique^7^,^24^,^25^. The film was first saturated by a magnetic field (=−10 mT) and then, a laser beam (=75 mW) was focused onto a small spot (5 μm in diameter) of the film under a reversed magnetic field (=3.3 mT) smaller than the coercive field (=8 mT). At this instant, the sample stage was moved along a desired direction, resulting in formation of a tilted straight DW. Alternatively, tilted DWs can be obtained from small arcs of larger circular domains.

### Experimental setup and measurement

The magnetic domain images were acquired using a magneto-optical Kerr effect (MOKE) microscope equipped with a charge-coupled device (CCD) camera. To apply the magnetic field onto the films, two electromagnets were attached to the sample stage. One of them was used to produce an in-plane magnetic field bias *H*_*x*_ up to 200 mT, whereas the other created an out-of-plane magnetic field *H*_*z*_ up to 35 mT. The possible effect from the small misalignment of the in-plane magnet as well as the ambient magnetic field is included in the *H*_*z*_ calibration, where this ambient magnetic field can be estimated by measuring the DW speed along right direction when +*H*_*x*_ is applied and the DW speed along left direction when −*H*_*x*_ is applied. This field difference between them is come from the small misalignment of the in-plane magnet. Using this system, the field-driven DW speed *v* was measured in the creep regime. To do so, a linear DW was initially placed at a tilted angle *θ*, as shown in the inset of Fig. 1, and then the DW displacement in the normal direction of the DW was monitored by the MOKE microscope under application of constant *H*_*z*_ and/or *H*_*x*_. The dependence of *v* on *H*_*x*_ exhibits asymmetries attributed to the variation of the DW energy density with *H*_*x*_, and the DMI-induced effective field can be directly quantified at a local minimum^11^.

## Additional Information

**How to cite this article**: Kim, D.-H. *et al*. Wide-Range Probing of Dzyaloshinskii–Moriya Interaction. *Sci. Rep.* **7**, 45498; doi: 10.1038/srep45498 (2017).

**Publisher's note:** Springer Nature remains neutral with regard to jurisdictional claims in published maps and institutional affiliations.
